# Recurrent Ileocolic Intussusception in Children: A Scoping Review

**DOI:** 10.1155/ijpe/8860000

**Published:** 2025-03-24

**Authors:** Salvatore Arena, Fabiola Cassaro, Giulia Maisano, Pietro Impellizzeri, Carmelo Romeo

**Affiliations:** Department of Human Pathology of Adult and Childhood “Gaetano Barresi”, University of Messina, Messina, Italy

**Keywords:** pathological leading point, pediatric patients, recurrence, recurrent intussusception

## Abstract

**Background:** Intussusception is the most common cause of acute intestinal obstruction in children. It can be initial idiopathic intussusception or a recurrent intussusception (RI), and in this latter case, there is not a validated algorithm for optimal treatment. The aim of the study is to review the international literature to evaluate the incidence of RI, to determine the rates of surgical intervention and pathological leading point (PLP), and to define the most appropriate management for children with RI. We included English-written papers with pediatric population, excluding case reports, papers with adult or mixed cases, studies focusing on ileo–ileal or colo–colic intussusception, meta-analysis studies, or papers with unclear or replaced data.

**Results:** A total number of 23 articles were included for a total of 26,731 patients affected by intussusception and 3164 recurrent patients (11.8%). The number of attempts of nonsurgical reduction ranged from 3 to 10 (median 5). On 2965 RI, 358 underwent surgery (12.1%). A pathologic leading point was found in 99 patients (3.95%).

**Conclusions:** The presence of a PLP does not seem to be associated with the recurrence of intussusception. More than 85% of RI underwent successful nonsurgical management. RI should be safely approached in the same way as primary intussusception, and surgery should be reserved to cases where a PLP has been suspected. In cases of multiple episodes, surgery can be considered an effective way to avoid recurrences, and this possibility should be discussed with parents.

## 1. Introduction

Intussusception is considered the most common cause of acute pediatric intestinal obstruction. This condition typically involves invagination of the ileum into the cecum or colon [1]. Although this pathology has been known for over two centuries, the etiology of intussusception in infants is still mostly unknown. Alternatively, intussusception can be induced by a pathological leading point (PLP), such as polyps, intestinal duplication, Meckel's diverticulum, or a malignant tumor—seen in approximately 10% of cases [2–4]. Even with the use of therapies such as hydrostatic, contrast, or air enema, for the management of uncomplicated intussusception, recurrence after nonoperative reduction remains relatively common, with recurrence rates ranging from 7% to 15% [5–8].

While the treatment algorithm for initial idiopathic intussusception is largely accepted, the management of recurrent intussusception remains controversial. Some authors prefer a surgical procedure after the first or at least second recurrence because a PLP is suspected [9–11]; others suggest treating recurrent intussusceptions with nonoperative reduction, similar to primary intussusception [12, 13].

The aim of the study was to review the international literature to evaluate the actual incidence of recurrence after primary intussusception, determine the rates of surgical intervention and PLP, and define the most appropriate management for children with recurrent intussusception.

## 2. Material and Methods

A scoping review of the literature was conducted following the PRISMA-Scr 2018 guidelines. A comprehensive literature search was performed in PubMed, with checks of relevant reference lists, using the search terms “recurrent intussusception” or “recurrence” and “intussusception” and “children” or “adolescents” or “pediatric patients.” The search was restricted to studies published in English between 1990 and August 2023. The inclusion criteria encompassed studies reporting data on pediatric patients diagnosed with intussusception, in which objective clinical outcomes were assessed. Articles were considered eligible if they provided information on recurrence rates, management strategies, and/or PLPs. Only original studies, including retrospective and prospective cohort studies, case–control studies, and randomized controlled trials were included. Exclusion criteria were applied to studies focusing on adult or mixed adult–pediatric populations, ileo–ileal or colo–colic intussusception, meta-analyses, systematic reviews, conference abstracts, and articles with unclear or incomplete data. Two examiners independently evaluated each paper, and disagreements were resolved through discussion with a third senior reviewer. The evidence level of each paper was rated according to Oxford Centre for Evidence-Based Medicine Levels of Evidence [14]. Full-text articles meeting the selection criteria were reviewed, and study data were abstracted in the same manner. All included studies were recorded in an electronic data sheet to analyze the study-level factors (country of origin and year of publication), procedure performed, number of patients, mean age, and outcome measures analyzed in each study. Statistical analyses were conducted using mean ± standard deviation where applicable, and studies with missing essential data were excluded from specific statistical evaluations.

## 3. Results

**Figure 1:**
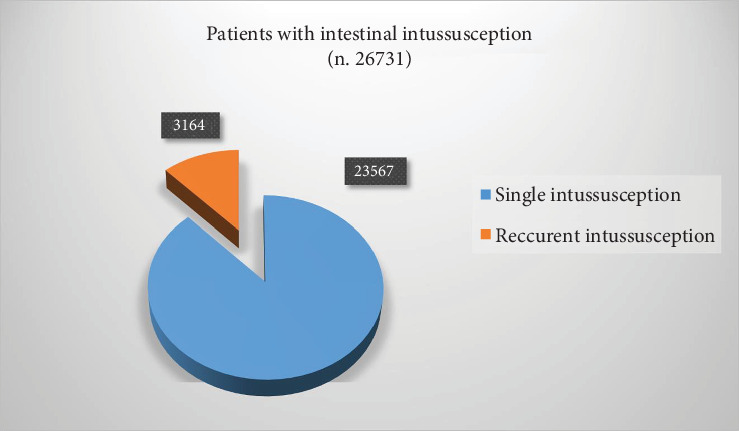
Distribution of patients with intestinal intussusception.

**Figure 2:**
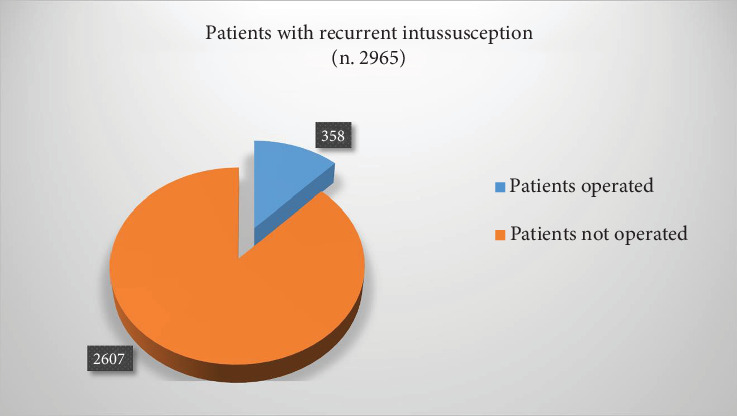
Distribution of patients with recurrent intestinal intussusception.

**Figure 3:**
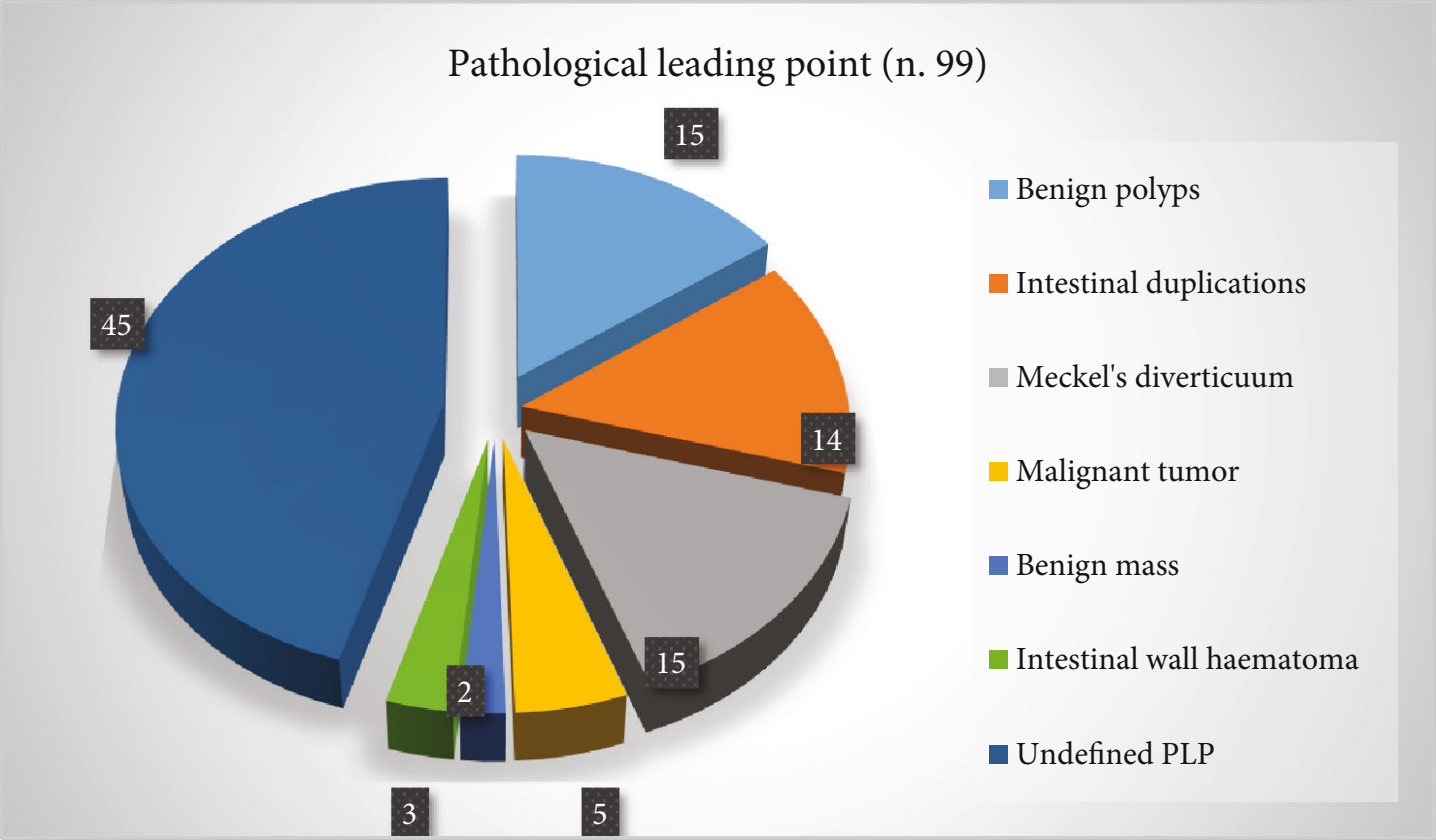
Distribution of pathological leading points in patients with intestinal intussusception.

Six hundred four papers were collected from PubMed. Five hundred eighty-one papers were excluded from the study because they were case reports (*n* 159) or discussed ileo–ileal intussusception (*n* 11) or were adult cases (*n* 78). Three hundred and thirty-three papers did not fit the aim. A total of 23 articles (Table 1) were retrieved for a total of 26,731 patients affected by intussusception and 3164 recurrent cases (11.8%) (Figure 1). Based on our data, 851 were males and 1713 females (male/female ratio 1.85/1). Of 2872 patients (data were not available for four papers) with RI, 4680 episodes of recurrence were reported; the average number of episodes was 1.63 for each case. The number of attempts of nonsurgical reduction ranged from 3 to 10 (5.33 ± 2.0, median 5). Of 2965 RI (data were not available for one paper), 358 underwent surgery (12.1%) (Figure 2). Among the 2505 RI (data were not available for two papers), a PLP has been found in 99 patients (3.95%). Specifically, 15 (15.1%) had benign polyps, 15 (15.1%) had Meckel's diverticulum, 14 (14.1%) had intestinal duplications, 5 had a malignant tumor (5.05%), 3 had an intestinal wall hematoma (3.0%), and 2 had a benign mass (2.0%). The remaining were undefined PLP (Figure 3).

## 4. Discussion

Recurrence of primary intussusception is considered a main complication following nonsurgical reduction, with an incidence of approximately 12% in over 20,000 reviewed cases. It has been observed that in addition to patient age, symptom duration, absence of vomiting, mass location in the right abdomen, and evidence of a PLP are all considered risk factors for recurrence [16, 32]. PLPs are Meckel's diverticulum, duplication of the ileum, polyps, tumors, and appendiceal stump. PLPs are associated with 0.3%–20% of cases of intestinal intussusception in children. Enlargement of mesenteric lymph nodes also appears to be a predictor of in-hospital intestinal intussusception recurrence (OR = 1.90). This result coincides with other studies that found that enlarged abdominal lymph nodes were related to the recurrence of intussusception. It was also observed that the incidence of lymph node enlargement was numerically higher in the recurrence group than in the nonrecurrence group [35].

Some scientific studies have considered other predictive factors of intussusception which may be different from those analyzed in previous studies [36–38]. Factors associated with recurrent intussusception included age (> 1 year), symptom duration (≤ 12 h), mass location (right abdomen), PLP, absence of bloody stool, or vomiting [24, 27]. These additional factors may provide a more complete understanding of potential disease predictors and help identify at-risk patients more accurately. Some authors located at the Children's Hospital of Los Angeles reported a 91% success rate following the first intussusception reduction attempt, with a recurrence rate of 8.5%. In the latter case, after nonoperative reduction techniques, different variables were highlighted including younger age, rectal bleeding, intestinal obstruction evident on imaging, or prolonged duration of symptoms [24, 39]. The same authors confirmed that the most common PLP was Meckel's diverticulum, followed by a juvenile polyp, Peutz–Jeghers syndrome, ischemic appendix, lymphoid hyperplasia of the terminal ileum, and enterocutaneous fistula. Male sex seems to play a role in the recurrence of intussusception, although there is little evidence about this factor [40].

Furthermore, among the included studies, Chen et al. [32] report an incidence of 1.28% under 2 years and 1.54% above 2 years; however, the same authors showed that the risk significantly increases after 5 years, reaching 8.1%. Similarly, Esmaeili-Dooki et al. [19] highlighted that the average age of patients with PLP is around 5 years (*p* = 0.08 compared to patients without PLP). Fisher et al. [29] found that the risk of PLP rises to 29% above 5 years, while Hsu et al. [20] reported a 36.4% risk in the same age group. These findings support the notion that when intussusception occurs beyond 5 years of age, the likelihood of PLP development is higher. By our review, it is unclear if the incidence of PLPs in recurrent intussusception is enhanced in older patients similar to the idiopathic condition. According to our data, in recurrent cases, the PLP rate was less than 5%, suggesting that cases with PLP are generally identified and diagnosed during the first intussusception episode [32]. Of all the PLPs, only five out of 99 were malignant tumors, specifically three unspecified ones and two Burkitt lymphomas. In recent literature, no statistically significant difference in age was found between recurrence and nonrecurrence groups [31]; an epidemiology report showed that the recurrence rate decreases from 10.1% to 5.3% in children over 3 years old [23]. A multivariable logistic regression analysis documented that an age of 2 years or older is an independent predictor of recurrence after successful air enema reduction [15, 17]. Xie et al. [41] also identified age > 2 years as a risk factor, in association with duration of symptoms > 48 h, rectal bleeding, and PLP, previously listed [25]. Moreover, reflecting the predominance of primary intussusception in males, our data documented also a male-to-female ratio of 1.85–1 [23, 31].

It is well accepted that the first-line treatment of idiopathic intussusception in hemodynamically stable patients, with an absence of signs of perforation or peritonitis, is nonoperative reduction [25, 28, 39]. Surgical intervention is limited to cases where nonoperative management is contraindicated when enema reduction is unsuccessful or in cases of hemodynamic instability. Conversely, management of recurrent intussusception proves challenging for pediatricians, radiologists, and pediatric surgeons [16]. It continues to be heavily debated, ranging from several repeated attempts of nonoperative intervention [18] to surgical exploration after at least one or two episodes, due to concerns of PLP presence [9–11].

In our retrospective study, we observed that on 2872 patients with RI, 4680 episodes of recurrence occurred with an average of 1.63 episodes per case and the number of nonoperative reduction attempts ranging from 3 to 10 (median 5). While some authors reported that the incidence of PLP is higher in children with multiple recurrences [29], others, such as Fisher et al. [29], showed that the rate of PLP was similar among patients with one, two, three, or more episodes of intussusception; Guo et al. [16] confirmed no significant differences in age, symptom duration, or paroxysmal crying between groups. A total of 45.5% of cases experienced one recurrence, while 54.5% had multiple recurrences. Univariate analysis found no significant differences in age, symptom duration, presence of blood in stool, paroxysmal crying, vomiting, mass location, or PLPs between single and multiple recurrence groups. Our data documented that the incidence of PLP on 2505 patients affected by RI was 3.95%, a value less than or equal to primary intussusception where the percentage of intussusception due to PLP is reported to be 6.5% [30]. Similarly to primary cases, it is noted that in RI, the incidence of PLP is higher in older patients, particularly in those older than 5 years, where it rises to 36% of cases [18, 22, 30]. Therefore, our data confirms that RI is not frequently related to the presence of PLP and can be largely considered idiopathic. Moreover, while it has been observed that the presence of PLP is not associated with the recurrence of intussusception [22], it has been argued that the presence of a PLP can be considered a cause of nonoperative reduction failure, requiring surgical procedure [26, 31]. For this reason, we supposed that only a minority of patients with PLP would have multiple episodes of RI and, consequently, the actual incidence of PLP in RI-affected patients does not seem to exceed that of patients with a single episode. According to this observation, this concern does not justify a surgical procedure. Our review showed that 2607 out of 2965 patients with RI (87.9%) were successfully managed nonoperatively, with no fatalities reported in any case. Therefore, our data suggest that the possibility of resolving an RI using enema is no less effective than in primary intussusception [42]. It has been supposed that in patients with RI, parental awareness of the clinical significance of the symptoms leads to a prompt hospital visit with less risk of a delayed diagnosis and subsequent need for surgery [21, 31, 34]. A few studies have analyzed intussusception reduction methods and their adequacy. The strategies utilized incorporate barium enema, air enema, and hydrostatic reduction with saline or other fluids. Reduction has been performed with or without sedation, with a few authors utilizing midazolam to improve patient tolerance [26, 34], whereas others performed the method on patients while awake [16, 29]. In a few studies, air enema has been shown to be more effective compared to barium enema and hydrostatic reduction [12, 16, 19, 26], whereas others prefer ultrasound-guided reduction citing a higher success rate and less complications [32, 34].

Based on our results, the possibility of PLP or multiple episodes of intussusception in cases of RI is of no higher risk to patients than in the case of the first episode. Although some authors reported that surgery can be considered following the third episode of RI due to the higher risk of subsequent recurrence [20], others successfully managed patients nonoperatively up to ten times [18]. However, because surgery substantially reduces the incidence of recurrence, it could be considered a valid choice to avoid hospital visits and parental concerns.

Limitations of the review are the lack of assessment of quality of the included studies with subsequent risk of bias and the inconsistency in providing strong recommendations during the decision-making process.

## 5. Conclusion

Our data suggest that RI should be safely approached in the same way as primary intussusception, and surgery should be reserved for cases where PLP has been suspected.

In our series, more than 85% of cases were resolved without surgical intervention. However, in cases of multiple episodes, surgery can be considered an effective way to avoid recurrences and subsequent hospital visits. Regardless, the possibility of surgery should be discussed with parents.

## Figures and Tables

**Table 1:** Summary of patient data included in the studies.

	**Patients with intussusception**	**Patients with RI**	**Surgical reductions in RI**	**No. of episodes of recurrence**	**No. of max attempts of nonsurgical reductions**	**Male/female**	**No. of pathological leading points**
Daneman et al. 1998 [7]	763	69	9	113	7	48/21	5
Trang et al. 2018 [15]	2223	254	8	380	7	151/103	3
Guo et al. 2017 [16]	1007	191	NA	481	6	117/74	19
Kim et al. 2017 [17]	454	32	2	NA	NA	NA	2
Esmaeili-Dooki et al. 2016 [18]	237	38	24	57	7	NA	1
Justice et al. 2010 [19]	598	85	3	125	10	NA	1
Niramis et al. 2010 [12]	1340	75	24	108	5	45/30	7
Hsu et al. 2012 [20]	672	85	30	119	5	NA	NA
Navarro et al. 2004 [13]	163	31	NA	56	5	NA	6
Ksia et al. 2013 [21]	505	28	4	39	3	17/11	1
Eshel et al. 1997 [22]	97	8	0	9	3	5/3	0
Chen et al. 2010 [23]	7541	597	64	676	NA	376/221	NA
Champoux et al. 1994 [24]	263	23	6	33	3	13/10	0
Talabi et al. 2018 [25]	51	3	0	5	3	NA	0
Özcan et al. 2016 [26]	401	41	5	NA	NA	33/8	1
Wang et al. 2015 [27]	2295	127	9	NA	NA	NA	3
Fecteau et al. 1996 [28]	258	28	37	13	4	NA	3
Fisher et al. 2015 [29]	666	96	20	155	NA	NA	6
Cho et al. 2019 [30]	491	68	20	113	7	46/22	5
Lee et al. 2018 [31]	137	23	1	23	NA	NA	0
Chen et al. 2021 [32]	5778	1158	94	2161	NA	777/381	26
Ma et al. 2020 [33]	683	115	14	NA	NA	78/37	2
Demirel et al. 2022 [34]	108	12	12	14	NA	7/5	8

## Data Availability

The data that support the findings of this study are available at https://unimeit-my.sharepoint.com/personal/salarena_unime_it/_layouts/15/onedrive.aspx?id=%252Fpersonal%252Fsalarena%255Funime%255Fit%252FDocuments%252Frecurrent%2520intussusception%26ga=1.

## General Statement

*Approval of the Research Protocol*. PROSPERO does not accept scoping reviews, literature reviews, or mapping reviews. This should not stop the authors from submitting their full protocol or completed review for publication in a journal.
